# Short- and Long-Range Connections Differentially Modulate the Dynamics and State of Small-World Networks

**DOI:** 10.3389/fncom.2021.783474

**Published:** 2022-01-25

**Authors:** Simon Arvin, Andreas Nørgaard Glud, Keisuke Yonehara

**Affiliations:** ^1^Department of Neurosurgery, Center for Experimental Neuroscience – CENSE, Institute of Clinical Medicine, Aarhus University Hospital, Aarhus C, Denmark; ^2^Department of Biomedicine, Danish Research Institute of Translational Neuroscience – DANDRITE, Nordic-EMBL Partnership for Molecular Medicine, Aarhus University, Aarhus C, Denmark; ^3^Multiscale Sensory Structure Laboratory, National Institute of Genetics, Mishima, Japan; ^4^Department of Genetics, The Graduate University for Advanced Studies (SOKENDAI), Mishima, Japan

**Keywords:** small-world, neuromodulation, neural oscillations, topology, simulation, network, criticality

## Abstract

The human brain contains billions of neurons that flexibly interconnect to support local and global computational spans. As neuronal activity propagates through the neural medium, it approaches a critical state hedged between ordered and disordered system regimes. Recent work demonstrates that this criticality coincides with the small-world topology, a network arrangement that accommodates both local (subcritical) and global (supercritical) system properties. On one hand, operating near criticality is thought to offer several neurocomputational advantages, e.g., high-dynamic range, efficient information capacity, and information transfer fidelity. On the other hand, aberrations from the critical state have been linked to diverse pathologies of the brain, such as post-traumatic epileptiform seizures and disorders of consciousness. Modulation of brain activity, through neuromodulation, presents an attractive mode of treatment to alleviate such neurological disorders, but a tractable neural framework is needed to facilitate clinical progress. Using a variation on the generative small-world model of Watts and Strogatz and Kuramoto's model of coupled oscillators, we show that the topological and dynamical properties of the small-world network are divided into two functional domains based on the range of connectivity, and that these domains play distinct roles in shaping the behavior of the critical state. We demonstrate that short-range network connections shape the dynamics of the system, e.g., its volatility and metastability, whereas long-range connections drive the system state, e.g., a seizure. Together, these findings lend support to combinatorial neuromodulation approaches that synergistically normalize the system dynamic while mobilizing the system state.

## Introduction

**Figure 1:**
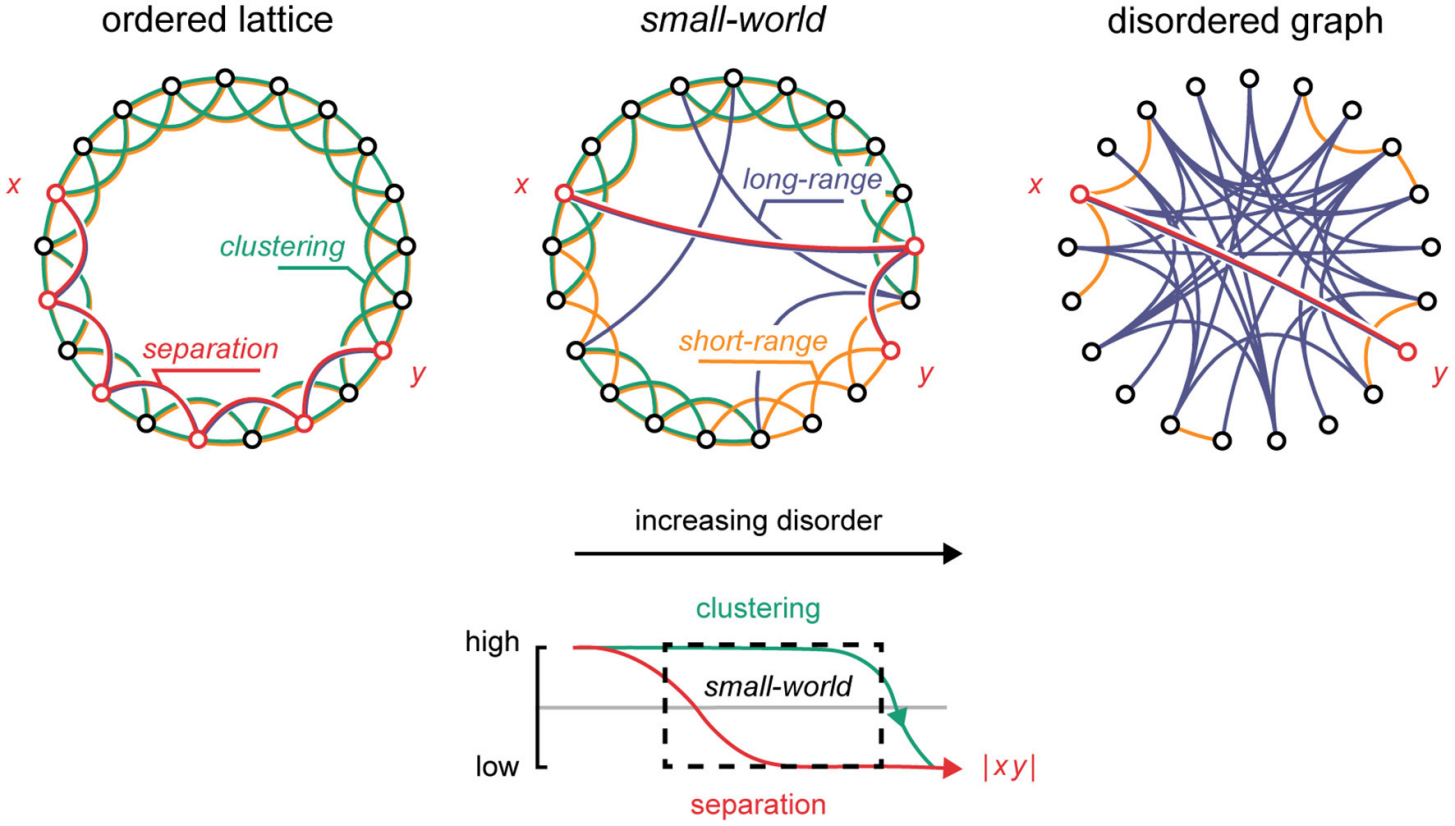
The small-world topology. Upper panel: By randomly rewiring an ordered lattice, it gradually transitions to a disordered graph. Within this transition, the small-world arrangement defines a critical state. Green: clusters. Yellow: short-range connections. Blue: long-range connections. Red: the shortest path between nodes *x* and *y*. Lower panel: with increasing randomness, the degree of separation between nodes in the network rapidly decreases (red), while clustering remains practically unchanged within a wide limit (green). The small-world topology corresponds to the dashed area containing high-clustering and low-separation properties.

The human brain is thought to contain billions of neurons that densely interconnect across short and long spatial distances (von Bartheld et al., 2016). The pattern of neuronal activity hinges on the anatomical and functional medium by which it is generated, and in which it propagates (Figure 1) (Wolfram, 1984a,b; Perc, 2007; Wang et al., 2010). In a hypothetical lattice, where nodes are highly ordered and hold no long-range shortcuts, signals tend to fizzle out locally due to the resistance that high-nodal separation exerts on global transmission (Shew and Plenz, 2012). This contrast with more disordered graphs where signals tend to overwhelm the global network span through dense interconnectivity. Intermediately, in the “*small-world”* network formed by integrating just a few long-range shortcuts into an otherwise ordered lattice (Watts and Strogatz, 1998), signals tend to reverberate, perched on the edge of chaos in a so-called “*critical”* state (Shew and Plenz, 2012; Kim and Lim, 2015). Intriguingly, it is thought that the functional topology of the brain tends to this criticality (Takagi, 2018), flexibly maneuvering it based on an immediate operational needs; by dynamically recruiting or abandoning short- and long-range functional connections, e.g., through coherence of neuroelectric oscillations (Singer, 1999; Buzsáki, 2006; Akam and Kullmann, 2014), or neuroplasticity (Dan and Poo, 2004; Shin and Kim, 2006), the brain maneuvers clustered and disordered topological phases tuned to local and global operational spans, respectively. Within this theoretical framework, the dynamics of the brain essentially reflect a dialectic on one hand pulling the brain to its topological extremes (Poil et al., 2012; Shew and Plenz, 2012; Hesse and Gross, 2014), while, on the other hand, keeping it near the critical state (Shin and Kim, 2006; Hesse and Gross, 2014; Priesemann, 2015; Takagi, 2018). Operating near criticality is thought to offer several neurocomputational advantages, e.g., high-dynamic range, efficient information capacity, and information transfer fidelity. In turn, aberrations from criticality have been theorized to underpin distinct neuropathologies, such as post-traumatic epilepsy and consciousness disorders (Colombo et al., 2016). Certainly, the malfunction of long- and short-range functional connections, by injury or otherwise, could have disastrous effects on the dynamics of the brain (Pevzner et al., 2016). In this *Original Research* article, we investigate specifically how long- and short-range connections affect the topological and dynamical properties of the small-world network. Our results indicate that short-range connections shape the *dynamics* of the system, whereas long-range connections define its *state*. We discuss the implications of these differential effects on clinical neuromodulation.

## Methods

See Table 1 for model parameters.

**Table 1:** Model parameters.

**Description**	**Notation**	**Notes**
**Network topology**
Short-range connections	h, H	[4–50]
Long-range connections	g, G	[0.001–10]
Adjacency matrix	A_mn_	
Small-world coefficient	ω	[−1–+1]
**Kuramoto-type simulation**
Oscillatory phase	*θ_*m*_*	[0°–360°]
Network synchrony	*r*	[0–1]
Natural frequency	ϵ	Gaussian: μ = 0, σ = 1
Total nodes	*N*	1,000

### Network Generation

To keep a manageable number of free parameters, and to reduce the artifacts of boundary conditions, we restricted our analysis to a generative ring network model based on the small-world model of Watts and Strogatz (1998). These ring networks were generated using custom Python code based on the open-source module *networkx*. Briefly, *N* = 1,000 nodes were each wired to their *h* nearest neighbors, thus denoted “*short-range*” connections (for *h* well below saturation, *h* < < *N*). Next, each node on average received an additional set of *g* random, yet unique, wires, which were denoted “*long-range*” connections (as wires of *g* did not equal those of *h*). Concretely, long-range connectivity *g* was generated by a nested loop given by *g* = *T*× *u*, where *T* is the maximum number of additional wires per node, and *u* is the average fraction of these actuated. To approximate biological gray-white matter ratios, while retaining a connected graph, we kept the range of short-range connectivity ~10 times that of long-range connections (Bajada et al., 2019; Mota et al., 2019).

Finally, each network was defined by its adjacency matrix, *A*_*mn*_, which was used for network simulation analysis (see Kuramoto's Model of Coupled Oscillators).

### Small-World Coefficient

To quantify the extent to which a network resembled a small-world network, we computed the small-world coefficient ω (Telesford et al., 2011). Essentially, the small-world coefficient compares the resemblance of a network to a perfectly ordered vs. a perfectly disordered arrangement based on the extent to which the nodes of the network are clustered and the extent to which they are separated. The small-world coefficient is defined as:


$$\begin{array}{l} \omega \;=\frac{L_{disordered}}{L}-\frac{C}{C_{ordered}}, \end{array}$$

where *L* is the average shortest path length between nodes in the network, and *C* is the degree of clustering (Figure 1). The disordered and ordered networks were generated based on the long-range connectivity given by *g* = *T*× *u* (see Network Generation). For the perfectly ordered network, no long-range connections were added, thus *u* = 0, and consequently *g* = 0. For the perfectly disordered network, the maximum number of long-range connections was introduced, thus *u* = 1, and consequently *g* = *T*.

The network parameters *C* and *L* were computed using common graph theory methods. Concretely, clustering *C* was computed as the network *transitivity*, such that:


$$\begin{array}{l} C=\frac{3\nabla }{Tr} \end{array}$$

where ∇ is the number of closed triplets in the network, and *Tr* is the maximum number of triplets. The average shortest path length *L* was given by:


$$\begin{array}{l} L=\;\underset{s,t\in V}{\sum }\frac{D\left(s,t\right)}{\;N\left(N-1\right)}, \end{array}$$

where *V* is the set of nodes in the network, *D(s,t)* is the shortest path length from node *s* to *t*, and *N* is the total number of nodes. Thus, when network separation *L* ≈ *L*_*disordered*_, and network clustering *C* < < *C*_*ordered*_, the small-world coefficient ω ≈ +1, meaning that the network approximates a perfectly disordered graph. Similarly, for the perfectly ordered lattice, when network clustering *C* ≈ *C*_*ordered*_ and network separation *L* >> *L*_*disordered*_, the small-world coefficient approximates ω ≈ −1. Crucially, the small-world topology is defined as the critical state possessing both qualities, namely, network clustering similar to an ordered lattice *C* ≈ *C*_*ordered*_,and network separation similar to a disordered graph *L* ≈ *L*_*disordered*_; thus, the small-world coefficient tends to ω ≈ *0* as the network tends to the critical small-world arrangement.

### Kuramoto's Model of Coupled Oscillators

Each node of the network was modeled as a coupled Kuramoto-type oscillator (Yamamoto et al., 2018), described by the set of *N*-coupled differential equations (Breakspear et al., 2010):


$$\begin{array}{l} {\overset{∙}{\theta }}_{n}=\varepsilon_{n}+\frac{K}{N}\overset{N}{\underset{m=1}{\sum }}A_{mn}\sin\left(\theta_{m}-\theta_{n}\right),\;n=1,\;\ldots ,\;N, \end{array}$$

where the *n*^th^ oscillator with a natural frequency ε_*n*_ adjusts its phase velocity ${\overset{∙}{\theta }}_{n}$ based on the pair-wise phase interactions with its coupled peers (provided by the adjacency matrix *A*_mn_, see Network Generation). The internodal coupling was *K* = 3, and the natural frequencies were distributed according to the Gaussian probability density with mean ε_0_ = 0. The state of the node (*n* = 1, …, *N*) was thus defined by its phase θ, which was calculated by the Livermore Solver for Ordinary Differential Equations (LSODA) method with a dynamic time step.

The degree of synchrony in the network was quantified by the order parameter *r*, given by:


$$\begin{array}{l} r\left(\theta_{m}\right)=re^{i\psi }=\frac{1}{N}\overset{N}{\underset{m=1}{\sum }}e^{i\theta_{m}}, \end{array}$$

where ψ is the mean phase of the set of oscillators *N*, and the scalar *r* represents the order, or phase uniformity, of the network. An open-source Python implementation of the Kuramoto oscillatory system is available online, which was used to generate the simulation data presented here (Damicelli, 2021).

### Stability and Attractiveness Analysis

To compute the *stability* of different network states, we set the initial synchrony level of the network *via* the initial nodal phases θ_*m*0_. Thus, for initial synchrony *r*_0_ = 0.5, on average 50% of the nodes of network had equal phases in the initial state. Then, at a predefined time-step Δ*t* = 250, not necessarily in the steady-state, the deviation of the network from the initial synchrony level was computed, revealing the stability of the initial state. Specifically, larger deviations reflect weaker stability. Repeating this process for all combinations of initial synchrony levels and connectivity parameters produces the stability heat maps depicted in **Figure 4A**.

To calculate the *attractiveness* of different network states, we checked which synchrony levels the networks shifted to during the simulations and scored the end synchrony level based on the size of the shift. For instance, for initial synchrony *r*_0_ = 0.5, and long-range connectivity *g* = 0.001, one network might end up in end synchrony level *r*_1_ = 0.0. This adds a score of *s* = *|r*_0_ –* r*_1_*|* = 0.5 to the end synchrony state *r*_1_. The synchrony states holding the highest cumulative scores had the highest attractiveness. Repeating this process for all the combinations of initial synchrony levels and connectivity parameters produces the attractiveness heat maps given in **Figure 4B**.

## Results

**Figure 2:**
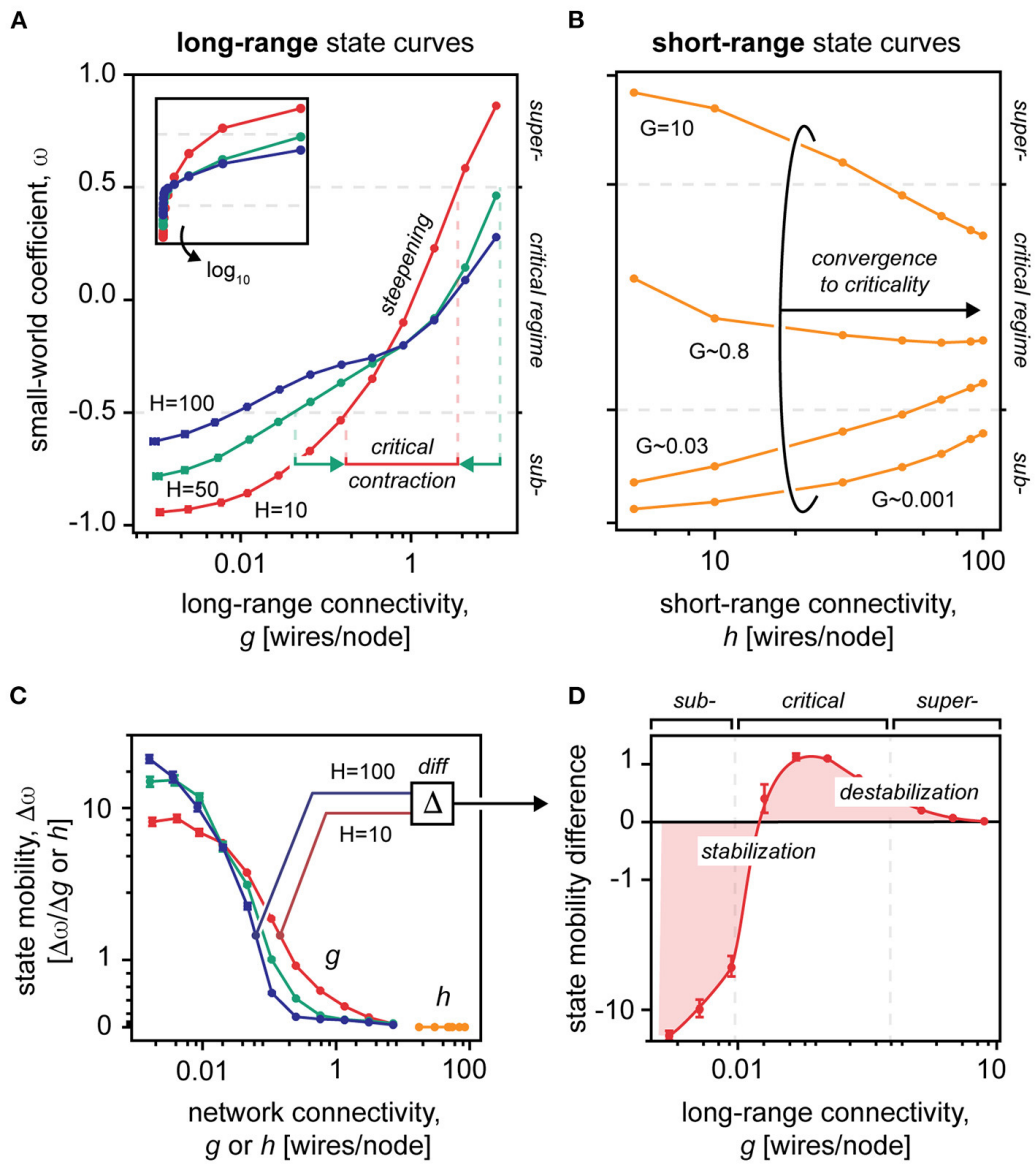
The small-worldness of networks with varying long- and short-range connectivity values. **(A)** Small-world coefficients in a *N* = 1,000 graph with static short-range connectivity *H* = 10, 50, 100 averaged over 100 samples, shown in semilogarithmic x-axis. Inset, shown in non-logarithmic x-axis. The vertical dotted lines represent the bounds of the critical regime for networks *H* = 10 and *H* = 100. Note that the criticality contracts as the static short-range connectivity decreases. **(B)** Small-world coefficients in a *N* = 1,000 graph with static long-range connectivity *G* (*G* ≈ 0.001, 0.03, 0.8, 10) averaged over 100 samples. Note that the short-range state curves converge to the critical state despite the underlying static long-range connectivity *G*. **(C)** The first derivative of the state curves, shown in A and B, constitutes state mobility of the network, i.e., how well it transitions from one topological state to another. Note that modulation of short-range connections *h* provides near null mobility of the topological state, vs. modulation of long-range connections *g*. Mobility of the topological state is mainly situated within subcritical and critical spaces, leaving near null mobility at high-connectivity values. **(D)** Difference in state mobility between networks with static short-range connectivity *H* = 10 and *H* = 100. The diagram shows that the topological state is stabilized in the subcritical space (negative values) and destabilized near criticality (positive values). Note that all plots have logarithmic x-axes. Data points are mean ± standard error of the mean.

To examine the effects of long- and short-range connections on the topological and dynamical properties of the small-world network, we applied a variation on small-world model of Watts and Strogatz (1998) and the Kuramoto model of coupled oscillators (Kuramoto, 1984). Concretely, we generated an ordered ring lattice consisting of *N* = 1,000 nodes, each node connected to its *h* nearest neighbors. To this base, we added, on average, *g unique* long-range connections per node. Thus, by definition, long-range connections *g* were topologically distinct from their short-range correlates *h*, holding true for short-range connectivity well below network saturation, *h* < < *N*. Then, to quantify the extent to which a network resembled a small-world network, we computed the small-world coefficient ω (Telesford et al., 2011). Essentially, the small-world coefficient compares the resemblance of a network to a perfectly ordered vs. a perfectly disordered arrangement based on the extent to which the nodes of the network are clustered and the extent to which they are separated. More specifically, ordered, *subcritical* lattices tend to ω ≈ –*1*, having high-clustering and high-separation parameters; disordered, *supercritical* graphs tend to ω ≈ +*1*, having low-clustering and low-separation parameters; and *critical* small-world topologies tend to ω ≈ *0*, having both the ordered and disordered tendencies balanced out (see Methods). Within this definition, we visualized the topological behavior of the network by plotting the small-world coefficient ω as a function of the long- and short-range connectivity *g* and *h*, respectively (Figure 2). Finally, we used the same topological framework to generate networks of coupled Kuramoto-type oscillators (Kuramoto, 1984).

### Long-Range Connections Dominate the Topological State

We first examined the roles of short- and long-range connections in defining the topological state of the network, specifically by keeping one parameter static (uppercase letters *H* and *G*) while modulating the other (lowercase letters *h* and *g*) (Figure 2). We found that modulations of the long-range connectivity *g* offered a near full topological range despite the underlying static short-range connectivity *H* (Figure 2A; ~70% ± 0.05; mean ± SEM). Yet, the opposite was not the case: Invariant to the underlying long-range connectivity *G*, increases to the short-range connectivity *h* all converged to the critical state (Figures 2A,B). In general, less than half of the topological range was attainable by short-range modulation alone. Thus, short-range connections appear to be poorly suited as a modulator of the topological state.

For further examination, we computed the first derivative of the topological state to reveal the *state mobility* Δω of the network, i.e., how readily the network moved from one topological state to another *via* changes to its connectivity parameters *h* and *g* (Figure 2C). We found that across all underlying long-range connectivities *G*, modulation of the short-range connectivity *h* had near null effects on the topological state. Modulation of the long-range connectivity *g* of the network, however, offered potent state mobilization within the subcritical and critical regimes, but near null mobility approaching supercriticality. The dominant role of long-range connections on the topological state was confirmed by dominance analysis (*R*^2^ ~ 0.648 for long-range vs. *R*^2^ ~ 0.003 for short-range connections).

These results together indicate that the topological state of the small-world network is dominantly defined by the long-range connectivity (Watts and Strogatz, 1998) and that the topological mobility of the network is the most potent well below supercriticality (Carhart-Harris et al., 2014).

### Short-Range Connections Shape the Topological Dynamics

Next, we evaluated how the underlying short-range connectivity *H* affects the topological behavior of the network, as reflected by the shape of the topological state curves (Figure 2). We found that as the static short-range connectivity *H* was reduced, the state curve steepened about the critical point, thus, contracting and “*right-shifting*” the critical regime to higher values of the long-range connectivity *g* (Figure 2A). This indicates that, to sustain the small-world criticality, poorly clustered networks (low *H*) must integrate long-range connections to a greater extent, yet within a narrower limit.

We then calculated the difference in state mobility of networks that had a high-static short-range connectivity (*H* = 100) and a low-static short-range connectivity (*H* = 10) (Figure 2C). In this difference plot, negative values reflect a reduction in the state mobility of the network, which essentially equates to a stabilization of the topological state (and oppositely for the positive values). Intriguingly, we found that, as the static short-range connectivity *H* was reduced, the stability of the topological state shifted to the subcritical regime, strongly destabilizing the small-world criticality (Figure 2D). This indicates that the short-range connectivity of the network has fundamental effects on the stability of the network across diverse topological regimes.

### Network Synchronizability

**Figure 3:**
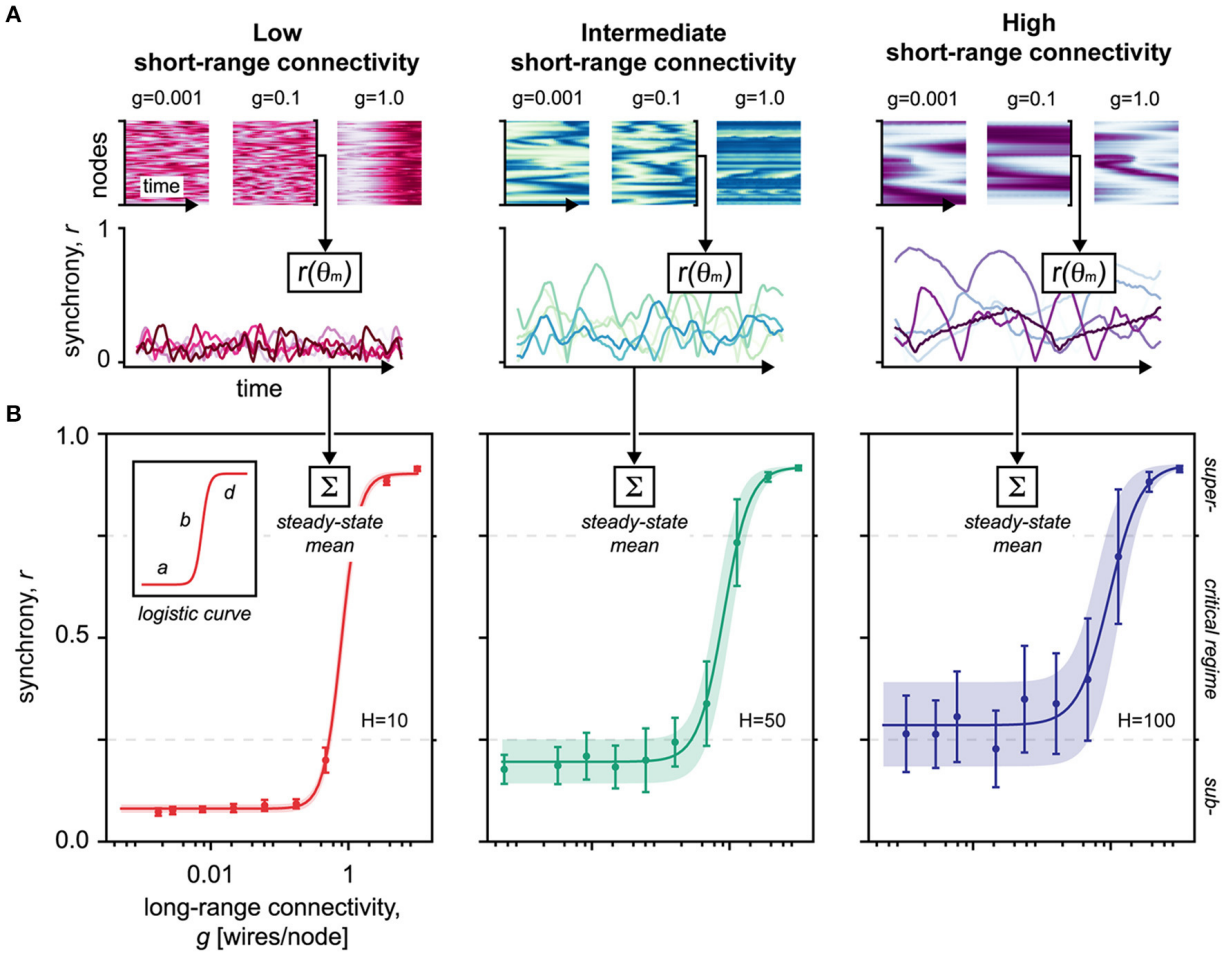
Simulations of Kuramoto's coupled oscillators in small-world networks. **(A)** Upper panel shows the network activity over time. Colormap represents the phase of the nodes (faint: hyperpolarized–strong: depolarized). *g*, long-range connectivity. Lower panel shows the global synchrony of the network over time. The network activity, sampled in the upper panel, was mapped by *r(*θ_*m*_*)* to reveal the network synchrony/order *r* given the set of oscillatory phases θ_*m*_. The lines represent individual trials, color-shaded to increase visual separability. Red, *H* = 10, low short-range connectivity. Green, *H* = 50, intermediate short-range connectivity. Blue, *H* = 100, high short-range connectivity. **(B)** Averaged over 15 trials, this diagram shows the steady-state synchronizability of the network as the long-range connectivity *g* increases. Data points are mean ± SD of the mean. The shaded area corresponds to the SD of the mean, fitted to the logistic function. The SD of the mean is equivalent to metastability of the network, defining a dynamical regime that facilitates flexible nodal interactions without stagnating in fixed positions (Hellyer et al., 2015). Logistic curve parameters: *a*, minimum synchronizability; *b*, critical slope; *d*, maximum synchronizability; *H*, short-range connectivity. Red, *H* = 10, low short-range connectivity. Green, *H* = 50, intermediate short-range connectivity. Blue, *H* = 100, high short-range connectivity.

To extend our topological findings, we examined the synchronization properties offered by small-world networks of varying short- and long-range connectivity parameters (Figure 3). To this end, we quantified the global network synchrony at the steady-state using Kuramoto's order parameter *r*, which reflects that the global phase uniformity of the network nodes (see Methods).

By gradually integrating long-range connections into the network structure, our simulations show that the synchronizability abruptly reaches a critical point at which the network shifts from a state of low synchrony to near-complete synchrony (Figure 3A). Such “explosive synchronization,” a critical transitioning, is characteristic for the Kuramoto-type coupled oscillators (Kuramoto, 1984; Gómez-Gardeñes et al., 2011; Boccaletti et al., 2016), and mirrors the topological criticality of small-world networks (Figure 1) (Watts and Strogatz, 1998).

Next, we modeled the synchronizability using four-parameter logistic regression (Figure 3B). Like in our topological findings, we found that the slope of the critical transitioning *b* steepened as the static short-range connectivity *H* was reduced, indicating a destabilization and narrowing of the critical regime (Figure 3B, inset). The minimal synchronizability *a* of the network moreover related proportionally to the static short-range connectivity, indicating baseline synchronization hinged separately on short-range interactions. Indeed, as more short-range connections are introduced, the network ultimately reaches a point of saturation where global synchronization becomes deterministic, invariant to topological modulations (Barahona and Pecora, 2002).

**Figure 4:**
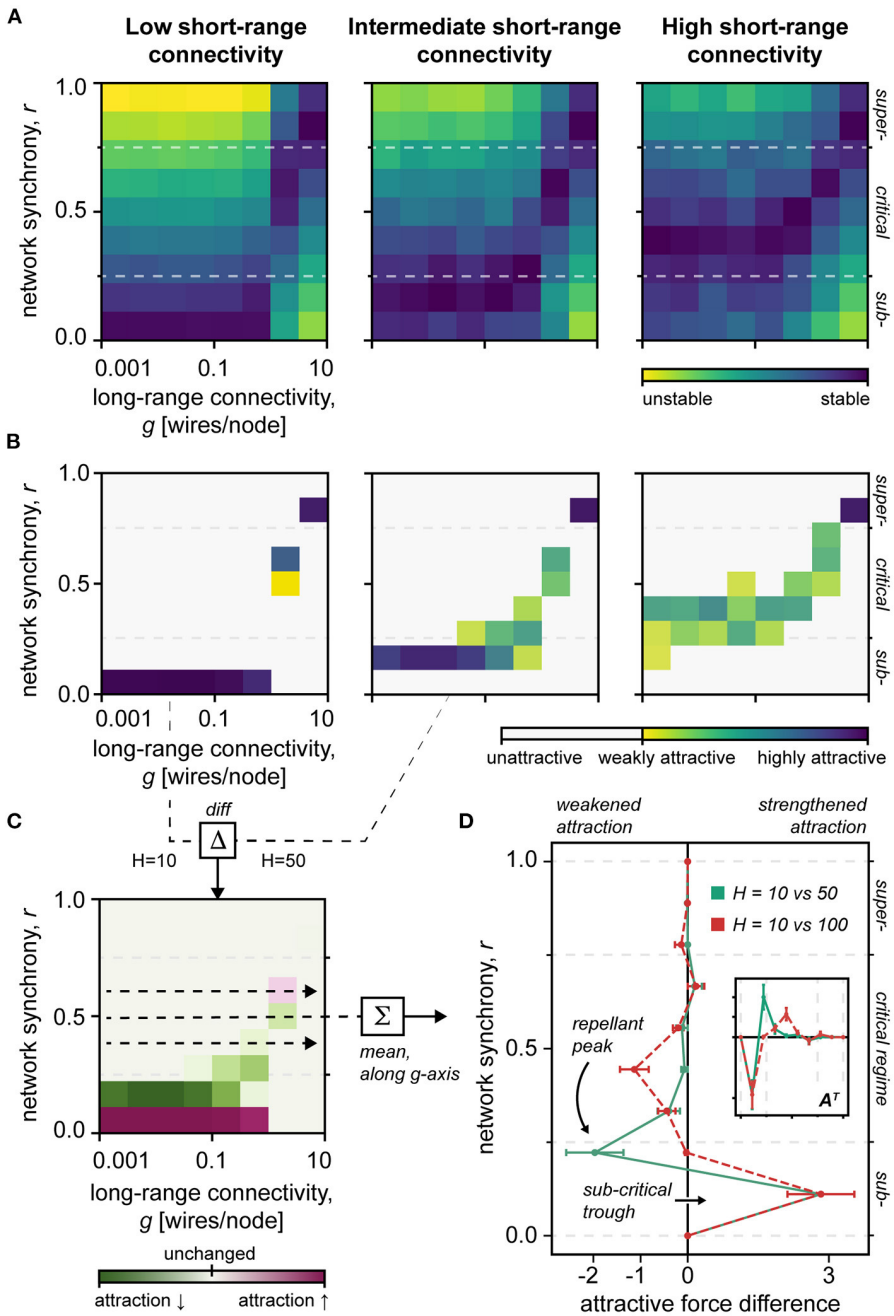
Network stability. **(A)** Heat map of the stability of different network states. Note the narrowing stability of the critical regime in the sparsely clustered network *H* = 10, vs. *H* = 50, 100. *H*, short-range connectivity; *g*, long-range connectivity; *r*, network synchrony; Blue, stable; Yellow, unstable. **(B)** Heat map of the attractiveness of different network states. Note the increased attractiveness of extreme network regimes, particularly subcriticality, in the sparsely clustered network *H* = 10. *H*, short-range connectivity; *g*, long-range connectivity; *r*, network synchrony; Blue, highly attractive; Yellow, less attractive; White, unattractive. **(C)** Difference in state attractiveness between low (*H* = 10) and intermediate (*H* = 50) short-range connectivity networks. *H*, short-range connectivity; *g*, long-range connectivity; *r*, network synchrony; Red, increased attractiveness; Green, decreased attractiveness; Faint, unchanged attractiveness. **(D)** The difference in attractiveness between differently clustered networks, meaned along the long-range connectivity *g*-axis. Note the repellant peak at the critical–subcritical boundary, and the subcritical trough, which together could facilitate subcritical entrapment. Inset shows the main plot data in a transposed view *A*^*T*^, which makes its similarity to the topological destabilization pattern clearer (Figure 2D). Red, dashed curve shows the attractiveness difference of sparsely clustered *H* = 10 and densely clustered *H* = 100 networks. Green, solid curve shows the attractiveness difference of sparsely clustered *H* = 10 and intermediately clustered *H* = 100 networks.

Finally, by calculating the divergence of the network from a predefined initial synchrony level (see Methods), we examined the stability and attractiveness of different network states (Figure 4). First, these data confirm that the stability of the critical regime narrows as short-range connectivity decreases. Second, as short-range connections are removed, we find that the network becomes increasingly attracted to subcritical synchrony states (Figure 4B), which aligns with the topological destabilization that favors subcriticality presented earlier (*cf*. red curves in Figures 2D, 4D, and inset).

### Network Metastability Depends on Short-Range Connections

Our data show that the functional interactions of the network converge as the static short-range connectivity decreases (Figure 3B, note narrowing standard deviation). Accordingly, we found that the long-range connectivity *g* in the poorly clustered network (low *H*) had a very high predictive power (PPS) on the global synchrony *r* of the network, whereas highly clustered networks (high *H*) were generally poorly predictable (*H* = 10, PPS = 0.93; *H* = 100, PPS = 0.43) (Wetschoreck et al., 2020). Furthermore, as the short-range connectivity of the networks tends to saturation (*H* to *N* = 1,000), the PPS drops to 0. More specifically, we find that the PPS is linearly proportional to the short-range connectivity of the network (PPS = −0.010H + 0.985;*R*^2^ = 0.99) (Supplementary Figure 1).

The PPS can be used to assess the *metastability* of the network. Thought to be inherent to cognition (Alderson et al., 2020), metastability defines a dynamical regime that accommodates flexible interactions of network nodes without stagnating in the fixed positions (Hellyer et al., 2015). Thus, our results show that metastability of the network depends linearly on the underlying short-range connectivity (by dominance analysis, *R*^2^ = 0.438 for the short-range vs. *R*^2^ = 0.136 for long-range connections). These simulation data altogether mirror our topological findings by suggesting that short-range connections are pivotal for the network's system dynamics (Figure 4).

## Discussion

We have investigated the effects of short- and long-range connections on the topological and dynamical properties of the small-world network. Converging with previous work (Watts and Strogatz, 1998), we demonstrate, first, that long-range connections determine the topological and functional state of the network. Second, we show that short-range connections shape the dynamics of the system, i.e., the stability of the system across diverse topological regimes (Figures 2, 4). Our findings together provide evidence that short- and long-range connections play distinct roles in shaping the behavior of the small-world network.

The topological properties of a network have fundamental effects on the activity taking place on it (Strogatz, 2001). Several works have, for instance, analyzed the spread of infectious disease in small-world networks, finding fluctuations between sporadic endemic and self-sustaining epidemic infectious cycles based on network disorder (Kuperman and Abramson, 2001; Rüdiger et al., 2020). Others have examined the synchronizability of coupled oscillators on small-world graphs (Barahona and Pecora, 2002; Nishikawa et al., 2003). Later, such simulations have been expanded to examine cortical oscillations and neuroplasticity (Maistrenko et al., 2007; Breakspear et al., 2010).

The human brain is a complex system sustained by the interactions of billions of neurons across local and global spatial scales. Previous work has shown that the functional topology of the brain tends to a small-world-like criticality that accommodates both local (subcritical) and global (supercritical) system properties (Bassett and Bullmore, 2017; Takagi, 2018). The hypothesis that the brain maintains a proximity to the critical state stems from the premise of superior computational adaptability to rapidly changing operational demands (Massobrio et al., 2015a). Contention posits, however, that signatures for criticality, e.g., power-law distributions, could be artifacts of sampling (Touboul and Destexhe, 2010; Marsili et al., 2013), multiplicative noise (Sornette, 1998) or emerge from “hidden variables” not necessarily linked to network topology (Aitchison et al., 2016; Morrell et al., 2021). While an exhaustive review is beyond the scope of this discussion (Beggs and Timme, 2012), we note that diverse data supports the relationship between critical neural dynamics and small-world topologies (Massobrio et al., 2015b; Tan and Cheong, 2017; Takagi, 2018) and the presence of critical signatures in human fMRI (Kitzbichler et al., 2009), local field potentials (Petermann et al., 2009), spike data (Friedman et al., 2012), human brain oscillations (Poil et al., 2008), and artificial neural networks (Shin and Kim, 2006). Indeed, congruent with a near-critical regime (Priesemann, 2015), the brain operates within a wide dynamic range that accommodate high-level cognition through global neural coordination (Taylor et al., 2015), and low-activity states, such as anesthesia (Brown et al., 2010), and, to some extent, sleeping (Priesemann et al., 2013; Tagliazucchi and van Someren, 2017), marked by weaker, more fragmented interactions outside the local milieu.

It is believed that neural oscillations, or “brain waves,” mediate short- and long-range neural connectivity through high- and low-frequency wavebands, respectively (Kopell et al., 2000; Buzsáki, 2006; Tiesinga and Sejnowski, 2009). In essence, the wave interference of oscillating neural populations facilitates the selective transfer of information (Singer, 1999; Buzsáki, 2006; Akam and Kullmann, 2010). Thus, it has been hypothesized that the malfunction of such neural interactions may have deleterious effects on the brain's system dynamics (Uhlhaas and Singer, 2006; Pevzner et al., 2016). In agreement with this premise, our results indicate that impairments to the network's short-range connectivity destabilize the small-world criticality in favor of extreme network regimes, i.e., sub- and supercriticality (Figures 2D, 4C). Such departure from criticality has been linked to large-scale fMRI signatures of unconsciousness (Tagliazucchi et al., 2016).

Subcritical networks tend to be states of desynchronization and clustering that perturb global network processing, e.g., cognition (Roozenbeek et al., 2013). Congruently, our simulations show that sparsely clustered networks, with poor short-range connectivity, exhibit weak metastability (Supplementary Figure 1), which has been correlated with deficits in cognitive flexibility (Hellyer et al., 2015).

Notably, our stability analysis indicates that damage to the short-range connectivity of the network could produce a “repellant peak” that effectively barricades the critical regime, trapping the network activity in a subcritical trough (Figure 4D). Such “subcritical entrapment” aligns with the behavioral heterogeneity of persistent disorders of consciousness (Giacino et al., 2014), e.g., partial retainment of cognitive processing, and lends theoretical support to the rehabilitation of the system dynamic, e.g., through short-range neural potentiation.

The supercritical network, on the other hand, tends to hypersynchrony, broadly resembling the state of seizures (Szaflarski et al., 2014; Zimmern, 2020). Indeed, researchers have argued that epileptiform seizures reflect a critical–supercritical transition (Arviv et al., 2016; Bauer et al., 2017; Freestone et al., 2017), which was recently supported by a strong electroencephalographic sign in human patients (Scheffer et al., 2009; Maturana et al., 2020). Similarly, Gerster et al. report that artificial neuronal oscillators on supercritical small-world graphs mirror electroencephalographic epileptic patterns (Gerster et al., 2020). The refractoriness of some types of epilepsy could thus reflect an underlying destabilization of the critical regime by elimination of the short-range connections, such as through cortical dysgenesis or brain trauma (Semah et al., 1998). Interestingly, recent work on the Kuramoto model has shown that generalized resource constraints seed the network to self-terminating supercritical episodes (Frolov and Hramov, 2021), consistent with epileptic recurrences.

One potential mechanism for the disruption of short-range neural connectivity may be an injury to key brain hubs that contain a high-cumulative weight of short-range connections (Gratton et al., 2012; Zhou et al., 2012; Haimovici et al., 2016; Yuan et al., 2017). Indeed, hubs, e.g., the cingulate cortex, have been shown to be instrumental for cognitive performance (Fagerholm et al., 2015; Li et al., 2019), and have profound effects on the functional connectivity of simulated networks (Aerts et al., 2016). It is interesting to note that compensation to injury could thus predictably be offered by the recruitment, or hyperactivity, of dense hub regions, which has been widely hypothesized (Hillary et al., 2011, 2015; Tang et al., 2012; Iraji et al., 2016), e.g., in components of the default mode network (Zhou et al., 2012).

Our findings altogether lend support to combinatorial neuromodulation strategies that target short- and long-range neural connectivity differentially, to normalize the system dynamic and mobilize the system state, respectively. Future work will target components of short- and long-range neural communication, e.g., through pharmacological neurostimulation *via* amantadine to preferentially enhance low-frequency brain oscillations (Ott et al., 2018; Ma and Zafonte, 2020), direct current stimulation of deep brain structures, e.g., hippocampal theta (Lee et al., 2013), or modulation of cerebral cortex gamma (Pink et al., 2019), e.g., using cell-type-specific optogenetic or pharmacogenetic modulation (Liu et al., 2020), or non-invasive transcranial magnetic stimulation at low frequencies (Farzan et al., 2012).

## Study Limitations

There are several limitations to this study. First, while providing a useful conceptual framework, Watts and Strogatz's ring model does not reflect real brain connectivity known to contain non-random edge distributions, e.g., “rich hubs” (van den Heuvel and Sporns, 2011), and a scale-free degree distribution (Eguíluz et al., 2005). Still, reduced topologies, e.g., generative small-worlds (Netoff et al., 2004; Perc, 2007; Tekin and Tagluk, 2017), and randomized graphs (van Vreeswijk and Sompolinsky, 1996; Tsodyks et al., 2000) remain valuable to neural network analysis by offering a controlled computational environment with manageable parameters and optimized network conditions.

Second, Kuramoto's oscillatory model represents a reduction of the complex interactions of distributed neural populations (Singer, 1999; Buzsáki, 2006). It is plausible that fuller physiological models would provide deeper insights into the precise mechanisms of such neural interactions. In support of the applicability of Kuramoto's equations, however, simulations have previously been applied to macaque (Honey and Sporns, 2008), and human brain research (Kitzbichler et al., 2009; Cabral et al., 2014), showing high congruence between simulation data and resting-state activity (Cabral et al., 2014; Vuksanović and Hövel, 2014). More broadly, reduced models (Siettos and Starke, 2016), such as two-state units (van Vreeswijk and Sompolinsky, 1996), and the *FitzHugh–Nagumo* model (Perc, 2007; Gerster et al., 2020), have been used extensively to examine complex network behaviors, such as self-organized balanced states (van Vreeswijk and Sompolinsky, 1996). Similarly, the abstraction offered by Kuramoto's model allows tractable simulations and analyses, holding high value for the investigation of more fundamental principles of oscillatory dynamics (Breakspear et al., 2010), such as the functional division of network connectivity examined here.

Despite these limitations, this study provides important insights into the relationship between network connectivity and critical system dynamics, which are broadly consistent with empirical reports and previous work (Haimovici et al., 2016). Future research should apply brain connectomic data and fuller network simulations to extend these findings.

## Data Availability Statement

The datatsets presented in this study can be found in the online repository: https://github.com/simonarvin/connectivity_smallworld.

## Author Contributions

SA and AG conceived the project. SA designed the project, performed the computations, and analyzed the data. SA, AG, and KY interpreted the data and wrote the manuscript. All authors contributed to the article and approved the submitted version.

## Funding

We acknowledge the following grants: Lundbeck Foundation (DANDRITE-R248-2016-2518; R344-2020-300; and R351-2020-1095), Novo Nordisk Foundation (NNF20OC0064395), and European Research Council Starting (638730) grants to KY.

## Conflict of Interest

The authors declare that the research was conducted in the absence of any commercial or financial relationships that could be construed as a potential conflict of interest.

## Publisher's Note

All claims expressed in this article are solely those of the authors and do not necessarily represent those of their affiliated organizations, or those of the publisher, the editors and the reviewers. Any product that may be evaluated in this article, or claim that may be made by its manufacturer, is not guaranteed or endorsed by the publisher.

## Supplementary Material

Supplementary Figure 1 Predictive power of long-range connectivity *g* on network synchrony *r*. The predictive power of long-range connections on the synchrony state of the network is linearly proportional to short-range connectivity of the network. In fact, as short-range connectivity *H* tends to saturation *N* = 1,000, the predictive power tends to 0. *g*, long-range connectivity; *r*, network synchrony; *H*, short-range connectivity; *N*, total nodes; *PPS*, predictive power.
