# Supplementary figures and images for: Short- and Long-Range Connections Differentially Modulate the Dynamics and State of Small-World Networks
Source: Front Comput Neurosci. 2022 Jan 25;15:783474. doi: 10.3389/fncom.2021.783474 (PMC8821822; doi:10.3389/fncom.2021.783474)

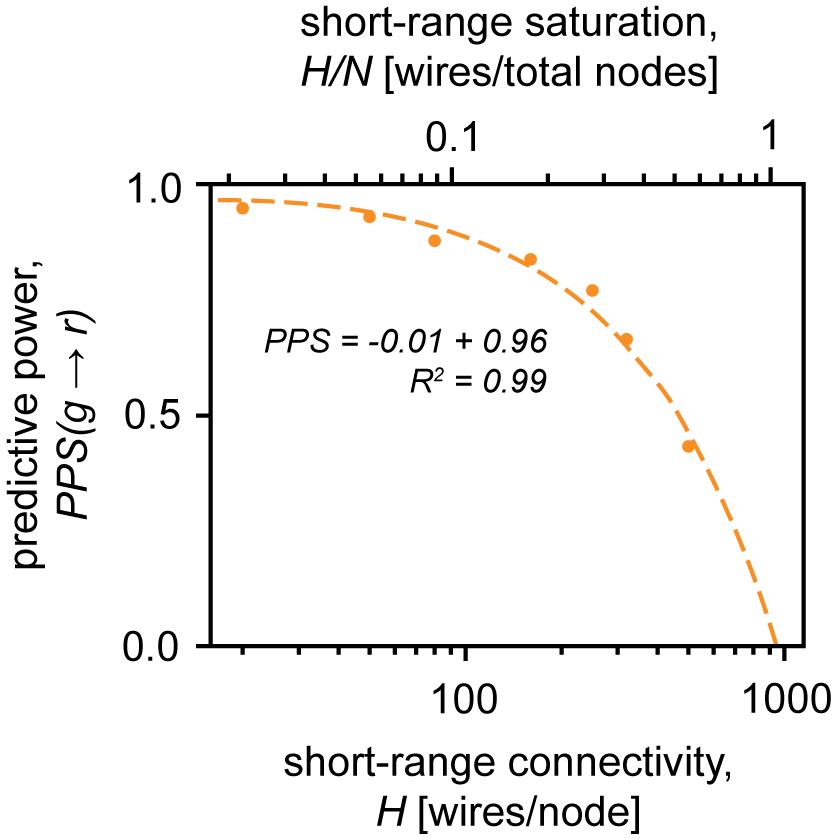

Supplement: Supplementary Figure 1 — Predictive power of long-range connectivity g on network synchrony r. The predictive power of long-range connections on the synchrony state of the network is linearly proportional to short-range connectivity of the network. In fact, as short-range connectivity H tends to saturation N = 1,000, the predictive power tends to 0. g, long-range connectivity; r, network synchrony; H, short-range connectivity; N, total nodes; PPS, predictive power. [file Image_1.tiff]
